# Ceftriaxone-resistant, multidrug-resistant *Neisseria gonorrhoeae* with a novel mosaic *penA-237.001* gene, France, June 2022

**DOI:** 10.2807/1560-7917.ES.2022.27.50.2200899

**Published:** 2022-12-15

**Authors:** Béatrice Berçot, François Caméléna, Manel Mérimèche, Susanne Jacobsson, Ghalia Sbaa, Mary Mainardis, Cyrille Valin, Jean-Michel Molina, Cécile Bébéar, Emilie Chazelle, Florence Lot, Daniel Golparian, Magnus Unemo

**Affiliations:** 1 Université Paris Cité, INSERM, IAME, Paris, France; 2 APHP, Infectious Agents Department, Saint Louis Hospital, Paris, France; 3 French National Reference Centre for bacterial STIs, Associated Laboratory for Gonococci, Paris, France; 4 WHO Collaborating Centre for Gonorrhoea and Other STIs, National Reference Laboratory for STIs, Department of Laboratory Medicine, Faculty of Medicine and Health, Ӧrebro University, Ӧrebro, Sweden; 5 Laboratoire Anse, Biogroup, Lyon, France; 6 AP-HP, Hôpital Saint-Louis, Lariboisière, Département de Maladies Infectieuses et Tropicales, Paris, France; 7 Université Paris Cité, UMR S976, INSERM, Paris, France; 8 University of Bordeaux, USC EA 3671, Mycoplasmal and Chlamydial Infections in Humans, Bordeaux University Hospital, French National Reference Centre for Bacterial STIs, Bordeaux, France; 9 Santé Publique France, French National Public Health Agency, Saint-Maurice, France; 10 Institute for Global Health, University College London, London, United Kingdom

**Keywords:** *Neisseria gonorrhoeae*, ceftriaxone, multidrug-resistant *N. gonorrhoeae* (MDR-NG), antimicrobial resistance, travel

## Abstract

We report a ceftriaxone-resistant, multidrug-resistant urogenital gonorrhoea case in a heterosexual woman in France, June 2022. The woman was successfully treated with azithromycin 2 g. She had unprotected sex with her regular partner, who developed urethritis following travel to Vietnam and Switzerland. Whole genome sequencing of the gonococcal isolate (F92) identified MLST ST1901, NG-STAR CC-199, and the novel mosaic *penA-237.001*, which caused ceftriaxone resistance. *penA-237.001* is 98.7% identical to *penA-60.001*, reported in various ceftriaxone-resistant strains, including the internationally spreading FC428 clone.

The emergence of ceftriaxone resistance in *Neisseria gonorrhoeae* (NG) has become a relevant concern worldwide. Very few new antibiotics are available for treatment of such cases. Here, we describe a case of local transmission in France following possible importation of a ceftriaxone-resistant Asian multidrug-resistant (MDR)-NG by the partner of the heterosexual case. 

## Clinical case description and diagnosis

In May 2022, a heterosexual woman in her late 40s with vaginal discharge consulted her general practitioner. Three days before symptom onset, she had vaginal unprotected sexual intercourse with her regular male partner, who had recently returned from business trips to Vietnam and Switzerland. The partner had urethritis symptoms, but he was not available for medical examination. The woman was screened (only vaginal swab) for *Neisseria gonorrhoeae* with a nucleic acid amplification test (NAAT) (Allplex STI Essential Assay Q, Seegene) and *N. gonorrhoeae* (NG) culture. The pathogen was detected by NAAT but viral culture was negative. Serological tests for HIV, hepatitis B virus/hepatitis C virus infections and syphilis were negative. The woman was empirically treated with a single intramuscular dose of ceftriaxone 1 g and, within one week, symptoms were resolved and NAAT was negative (test of cure was negative 7 days post-treatment). However, approximately 6 weeks later, in June 2022, the woman returned with vaginal discharge, after having sexual intercourse only with her partner. The NAAT and culture of vaginal swabs were NG-positive. Extragenital sites were not screened as the woman reported no oral/anal sex. Previous histories of STIs in the woman and her partner were not known.

Antimicrobial susceptibility testing was performed on the vaginal sample. No empirical treatment was given before antimicrobial susceptibility testing results were available. Results revealed that the isolate (referred to as F92) was resistant to ceftriaxone and susceptible to azithromycin. Thus, the general practitioner did not administer ceftriaxone and, accordingly, only a single oral dose of azithromycin 2 g was given, which successfully treated the infection. Tests of cure performed 2 weeks post-treatment, including NAATs on vaginal, anorectal and oropharyngeal swabs and culture, were negative.

## Antimicrobial susceptibility testing

The F92 isolate was cultured from a vaginal swab on PolyViteX agar (bioMérieux) in a humid 5% CO_2_-enriched atmosphere for 24 h at + 36 ± 1°C. Species verification was performed by matrix-assisted laser desorption/ionisation-time of flight (MALDI-TOF) mass spectrometry (Vitek MS, BioMérieux). Minimum inhibitory concentrations (MICs) were determined by Etest (BioMérieux) for 12 antimicrobial drugs and interpreted using European Committee on Antimicrobial Susceptibility Testing (EUCAST) recommendations [1], and by agar dilution for novel antimicrobials zoliflodacin [2], gepotidacin [3], and lefamulin [4]. The F92 isolate was resistant to ceftriaxone and other extended-spectrum cephalosporins (ESCs), benzylpenicillin, ciprofloxacin, and tetracycline, but susceptible to azithromycin and spectinomycin. The MICs of gentamicin, rifampicin and ertapenem as well as zoliflodacin, gepotidacin and lefamulin were considered wild-type according to previous publications and because no known antimicrobial resistance determinants for these antimicrobials were detected (Table).

**Table:** Minimum inhibitory concentrations of 15 antimicrobial drugs for the ceftriaxone-resistant, multidrug-resistant *Neisseria gonorrhoeae* F92 isolate, France, June 2022

Antimicrobial drug	MIC in mg/L	Interpretation (EUCAST v 12.0 [1])
Ceftriaxone	0.5	Resistant
Cefixime	2	Resistant
Cefotaxime	2	Resistant
Cefoxitin	2	Resistant
Benzylpenicillin	2	Resistant
Ciprofloxacin	> 32	Resistant
Tetracycline	4	Resistant
Azithromycin	0.5	Susceptible
Spectinomycin	16	Susceptible
Gentamicin	8	NA (Wild-type MIC)
Rifampicin	0.5	NA (Wild-type MIC)
Ertapenem	0.032	NA (Wild-type MIC)
Zoliflodacin^a^	0.064	NA (Wild-type MIC)
Gepotidacin^a^	0.25	NA (Wild-type MIC)
Lefamulin^b^	0.5	NA (Wild-type MIC)

EUCAST: European Committee on Antimicrobial Susceptibility Testing; MIC: minimum inhibitory concentration; NA: not applicable (because of lack of breakpoints for interpretation).

^a^ Pre-licensing international phase III randomised clinical trials underway [2,3].

^b^ Novel antimicrobial that has shown promising results in MIC testing [4].

Antimicrobial MIC in mg/L interpretation (EUCAST v 12.0 [1]). Wild-type MIC is the MIC usually observed for *Neisseria gonorrhoeae* not resistant to the antibiotic tested.

## Molecular investigation

Genomic DNA from F92 was isolated using QIAsymphony (QIAGEN) with the DSP DNA Mini Kit (QIAGEN). Whole genome sequencing (WGS) and bioinformatic analysis were performed as described [5,6], and sequencing reads are available at DNA Data Bank of Japan (DDBJ)/European Nucleotide Archive (ENA)/GenBank (Accession number: JAPIVM000000000).

F92 belonged to multilocus sequence typing (MLST) sequence type (ST) 1901, a new *N. gonorrhoeae* multi-antigen sequence typing (NG-MAST) ST (*porB-*2553, *tbpB-*2459), and the novel *N. gonorrhoeae* sequence typing for antimicrobial resistance (NG-STAR) ST4837, because of the new *penA* allele, in the NG-STAR clonal complex 199 (CC199). Resistance to ESCs was conferred by the novel mosaic *penA* allele designated as *penA-237.001*, encoding an amino acid sequence 99.1% identical (i.e. five substitutions: P328A, A480P, S483T, I485T and T534A; nucleotide identity: 98.7%) to that encoded by *penA-60.001*, which causes ESC resistance in the internationally spreading FC428 clone [5,7-12]. The *penA-237.001* encodes a mosaic penicillin-binding protein 2 (PBP2) including the amino acid substitutions A311V, I312M, V316T and G545S associated with ESC resistance [13]. One NG isolate with *penA-237.001* (isolate UK722) was recently isolated from a female patient described in the United Kingdom [12]. Many additional resistance determinants generated the multidrug-resistant (MDR) phenotype (Table), e.g. ‘A’ deletion in the *mtrR* promoter, PorB G120K/A121D, GyrA S91F/D95A, ParC S87R and *rpsJ* V57M [13].

**Figure 1:**
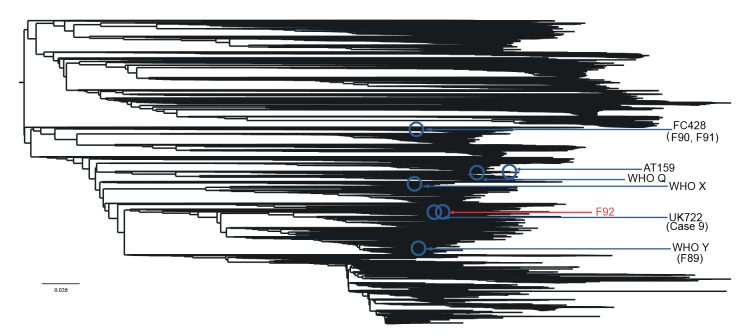
Core phylogenomics of publicly available *Neisseria gonorrhoeae* genome sequences (n = 36,310) showing the ceftriaxone-resistant, multidrug-resistant F92 gonococcal isolate from France and other published ceftriaxone-resistant gonococcal strains (n = 28), June 2022

**Figure 2:**
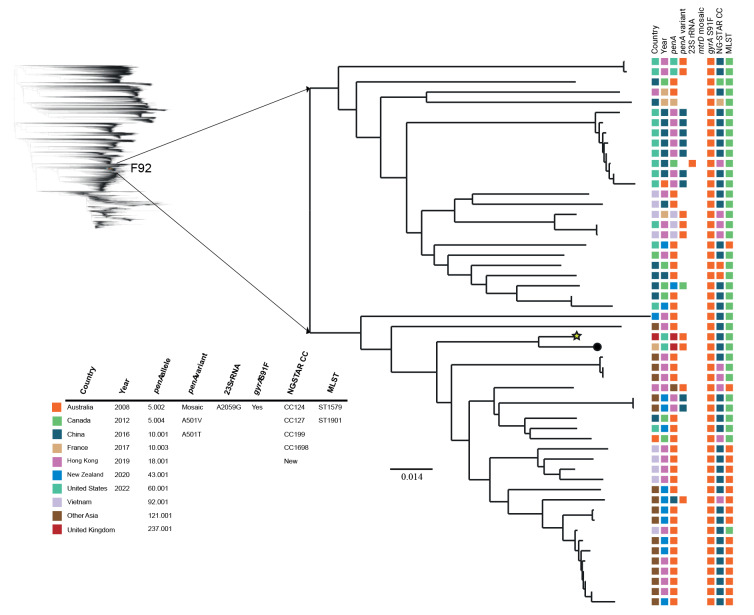
Phylogeny of the most closely related publicly available *Neisseria gonorrhoeae* genome sequences (n = 52) and the ceftriaxone-resistant, multidrug-resistant F92 gonococcal isolate from France, June 2022

CC: clonal complex; NG-STAR: *Neisseria gonorrhoeae* sequence typing for antimicrobial resistance; MLST: multilocus sequence typing.

The F92 isolate has 2,546, 5,152, and 5,068 single nucleotide polymorphism (SNP) differences compared with the ceftriaxone-resistant NG isolates F89, F90 and F91 obtained in France in 2010, 2017, and 2019, respectively [5,7,14]. However, F92 is closely related to the recently reported UK722 isolate described in the United Kingdom (Case 9) [12] with identical genotypes and only 42 SNP differences. Phylogenomic comparison to other gonococcal genomes (n = 36,310) showed that F92 belonged to the genomic sublineage A2, where ceftriaxone-resistant isolates have been previously identified. However, the ceftriaxone-resistant MDR F92 isolate was very distant from the internationally spreading FC428 clone [8-13] (Figure 1). F92 instead clustered with several of the mostly recently detected Asian MLST ST1901 and ST1579 isolates (Figure 2); the three most similar isolates (MLST ST1901) differed by 1,381–1,392 SNPs. The majority of these NG isolates were susceptible to ceftriaxone as they harboured non-mosaic *penA-5.002* (Figure 2).

The F92 isolate from France (red arrow) is compared with other ceftriaxone-resistant gonococcal strains (blue arrows and circles), among publicly available gonococcal genomes (n = 36,310) downloaded from the European Nucleotide Archive using the search term ’Taxon:485’ (on 4 August 2022). The internationally spreading ceftriaxone-resistant FC428 clade (including isolates F90 and F91) [5,7-12], the closely related UK722 isolate [12], the first described ceftriaxone-resistant extensively drug-resistant WHO X [21] and WHO Y (F89) [14] strains, and the first two strains with ceftriaxone resistance combined with high-level azithromycin resistance, i.e. AT159 [6] and WHO Q [22], are shown.

Publicly available gonococcal genomes (n = 36,310) were downloaded from the European Nucleotide Archive using the search term ’Taxon:485’ (on 4 August 2022) and analysed as previously described [23]. Briefly, all genomes were characterised based on a core genome MLST (cgMLST) scheme defined by the 2016 WHO gonococcal isolates, WHO Q and WHO R using Ridom Seqsphere + (v.8.4.1). Pairwise comparisons of the cgMLST alleles (n = 2,062) were performed and subsequent neighbour-joining tree was constructed in Ridom Seqsphere + (v.8.4.1). Phylogenomic trees and subtrees were visualised in Microreact (https://microreact.org). Additionally, closely related isolates (n = 52) to F92 (black solid circle), including the closely related UK722 isolate [12] (star) with 42 SNP differences, were characterised for antimicrobial resistance determinants and typing schemes using CLC Genomics Workbench (v22.0.1) as previously described [6,23].

## Discussion

The resistance to ESCs in NG has been low in France (0.8% and 0.2% of cefixime-resistant isolates in 2019 and 2021, respectively) [15] and Europe (0.8% of cefixime-resistant isolates in 2019) [16], since the decline of the widespread MLST ST1901, NG-MAST ST1407 clone [17]. Furthermore, the high-level ceftriaxone-resistant F89 isolate, which emerged in this genomic background, was reported only in France and Spain [14] and its spread was likely limited by a decreased biofitness [18]. The ceftriaxone-resistant F90 and F91 isolates, associated with the internationally spreading FC428 clone containing *penA-60* [5,7-12], appeared in France in 2017 and 2019, respectively [5,7]. Notably, the patient with F91 was infected by her husband, who acquired the infection from a sex worker in Cambodia. FC428 has an adequate biofitness [10]. To our knowledge, the F91 has not yet spread further within France.

The assumed importation of MDR-NG to Europe from Asia, where ESC resistance has been reported to be high and increasing [11,13,19], is worrying. The described isolate is ceftriaxone-resistant because of the mosaic *penA-237.001*, which closely resembles *penA-60.001*. The genome of this MLST ST1901 strain is genomically distant from the cefixime-resistant MLST ST1901 clone that has caused ESC-resistance – especially in Europe – for over a decade [17]. However, it is similar to recently described Asian ceftriaxone-susceptible isolates carrying a non-mosaic *penA-5.002* [20]. Accordingly, the F92 isolate with a novel mosaic *penA-237.001* causing ceftriaxone resistance is unique and it may result from a horizontal gene transfer of *penA-237.001* from commensal *Neisseria* species to a NG MLST ST1901 strain with *penA-5.002* in Asia. A limitation of our report is that no details were available from the partner, and no strain was available for analysis.

## Conclusions

The international emergence and transmission of NG isolates with resistance to ceftriaxone, which is the last remaining option for first-line gonorrhoea treatment, is a public health concern. Increased awareness of sporadic ceftriaxone-resistant cases and improved prevention, early diagnosis (including contact tracing and treatment of index case and contact) and surveillance of antimicrobial resistance, including test of cure and genome sequencing, are crucial to improve prevention, early diagnosis and surveillance. We show that the new antimicrobials zoliflodacin, gepotidacin and lefamulin all have a high in vitro activity also against the sporadic ceftriaxone-resistant strains. Indeed, novel effective and affordable antimicrobials for treatment of gonorrhoea and, ultimately, an effective gonococcal vaccine are needed.
